# Heart rate and blood lactate responses during the volleyball match

**DOI:** 10.1038/s41598-022-19687-3

**Published:** 2022-09-12

**Authors:** Cengiz Akarçeşme, Elif Cengizel, Ömer Şenel, İbrahim Yıldıran, Zeki Akyildiz, Hadi Nobari

**Affiliations:** 1 Department of Coaching Education, Faculty of Sport Sciences, Gazi University, Ankara, Turkey; 2 Department of Physical Education and Sports, Faculty of Sport Sciences, Gazi University, Ankara, Turkey; 3 Department of Exercise Physiology, Faculty of Educational Sciences and Psychology, University of Mohaghegh Ardabili, Ardabil, 5619911367 Iran; 4 Department of Motor Performance, Faculty of Physical Education and Mountain Sports, Transilvania University of Braşov, Braşov, 500068 Romania; 5 Faculty of Sport Sciences, University of Extremadura, Cáceres, 10003 Spain

**Keywords:** Health occupations, Engineering, Mathematics and computing

## Abstract

This study aimed to examine the heart rate and blood lactate responses of female volleyball players during the match according to the player positions. A total of 24 senior female volleyball players (middle blocker (n = 6), setter (n = 6), spiker (n = 6), and libero (n = 6)) were monitored for heart rate and blood lactate before, during and after a volleyball match. The mean heart rate and blood lactate level of volleyball players were determined 117.2 ± 13.9 bpm, 2.7 ± 1.2 mmol/L during the match. Heart rate was statistically different between all playing positions except middle blockers vs. spikers (p < 0.05). The blood lactate levels between the groups were not statistically different. The setters have the lowest heart rate and the libero players have the highest during the match. All subjects have a heart rate 50–60% and below 50% of their heart rate maximum during more than half of the match duration. These current results can be used by coaches to determine a specific training load based on the heart rate and blood lactate differences between playing positions.

## Introduction

Volleyball is an intermittent sport that often includes short bouts of high-intensity activity followed by periods of low-intensity activity^1–3^. The total duration of a match—about 60–90 min^4^—and the high-intensity bouts of exercises require volleyball players to have a well-developed aerobic and anaerobic energy system^2,5^.

Although it is reported the rest interval was 2.5 times the duration of the rally during the competition^6^; work:rest (W:R) ratio in high level senior male volleyball was 1:5.8 s (rally length: 4.99 ± 4.35 s; rest time: 29.02 ± 19.44 s^7^), and W:R ratio in high level senior female volleyball was 1:4.20 s (mean rally length 6.88 ± 5.94 s and rest time 28.92 ± 18.21 s^8^). These ratios indicate that volleyball is a sport which is characterized by diverse movements and energy levels which are interchanged during the match and which require energy from both aerobic and anaerobic metabolism^9–12^ and in training it is suggested to perform the activities which use ATP-CP system as the source of energy^6^.

Although it is a team sport as in other sports, the specialized playing positions in volleyball enable the athletes to perform different technical and tactical tasks. The work load which is specifically determined for each player position brings along the differentiation in physiological loading as well. For instance, based on the fact that the setters run to the long distance balls to setting and therefore cover longer distances than the spikers; Nikolaidis et al.^13^ predicted that setters must have a higher aerobic capacity than spikers. In the training of the athletes, it is necessary to determine this load and to know what kinds of demands are placed by the matches on each player^14^.

As required by the nature of the match and since it is dynamic and continuously volatile, it sounds impossible to understand and identify all mechanisms performed by the players during a volleyball match. There are lots of laboratory tests performed for determining the mental and physical structures of the players^15^. Furthermore, the researchers perform studies in numerous sports disciplines that determine heart rate (HR) and blood lactate (La) levels to obtain the physiological responses of the athletes within the competition environment^16–20^. However, especially in volleyball, there is limited number of studies that investigate the HR and blood La levels of the players before, during and after the match^9,15^. On the other hand, just as it is suggested to design the training sessions considering the W:R ratio during the competition^7^, it is also essential to determine the physiological responses of the players during this time period such as HR and blood La, and to include them in the match-specific training design. It is a matter of questioning how the physiological load in the match progresses according to the player positions. We hypothesized the heart rate and blood La responses would be different for the positions of libero, outside hitter, middle blocker, and setter during the volleyball match. Therefore, the purpose of this study is to determine the HR and blood La responses in elite female volleyball players before, during, and after the match according to playing positions.

## Material and methods

### Participants

Twenty four elite female volleyball players competing in the Turkish Volleyball Super League and 1st League who are to be chosen up to the National Team have participated in this study voluntarily. The subjects were divided into four groups as middle blockers (n = 6), setters (n = 6), spikers (n = 6) and liberos (n = 6, Table 1). All participants were informed of the risks and benefits of participating in the research and provided informed consent before the measurements. The study was performed in accordance with the Declaration of Helsinki and approved by the Local Ethics Committee of the Gazi University (No: 2022-101). Participants (a) under the age of 18 and (b) who had a disability or surgery in the last 6 months were not included in the study.

**Table 1:** Characteristics of the subjects (n = 24).

	Setter	Spiker	Middle blocker	Libero
Age (year)	22.2 ± 2.6	21.8 ± 1.5	22.5 ± 2.0	20.5 ± 1.7
Training experience (year)	12.5 ± 4.8	10.3 ± 3.6	10.5 ± 3.2	9.0 ± 1.8
Body height (cm)	180.2 ± 2.6	183.7 ± 6.0	183.8 ± 4.8	172.0 ± 6.7
Body mass (kg)	61.3 ± 5.2	67.6 ± 2.9	68.5 ± 7.6	59.5 ± 1.9
BMI (kg/m^2^)	18.8 ± 1.8	20.1 ± 2.0	20.1 ± 1.5	20.1 ± 0.9
RHR (bpm)	62.1 ± 4.3	64.3 ± 4.3	63.2 ± 4.5	64.0 ± 3.7

*BMI* body mass index, *RHR* resting heart rate.

### Design and procedures

Within the scope of this study, 3 friendship matches (3 sets per each match) were played between the equal level teams and it has been endeavoured to designate the match conditions in compliance with the real playing environment. For this purpose, referees were appointed and match rules (warm-up, set beginning and ending, technical time-outs) were applied during the match. In each match the HR of 8 of the players were tracked simultaneously by telemetric method and their blood La samples were taken. During this period, the players who were subject to measurement stayed in the match for 3 sets within the conditions required by their positions; and no players within the match were replaced on the court by a substitution, except for the rotation between middle blockers and liberos at the back row position.

**Figure 1:**
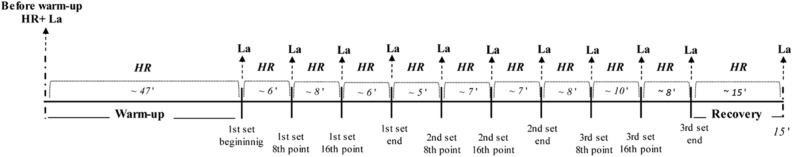
Timeline and data collection.

A total of 12 blood samples (0.5 µl) were taken from the subjects before the competition (before the warm-up) after the warm-up (just before the start of 1st set), during 1st set 1st technical time-out, during 1st set 2nd technical time-out, at the end of the 1st set, during 2nd set 1st technical time-out, during 2nd set 2nd technical time-out, at the end of the 2nd set, during 3rd set 1st technical time-out, during 3rd set 2nd technical time-out, at the end of the 3rd set, and 15 min after the end of the match. These samples were taken from the subjects with the help of a lancet by piercing a hole at their earlobes. The convenient blood samples were transferred to the disposable test strips and were analysed using a portable La analyser (Lactate Scout, SensLab GmbH, Leipzig, Germany). The blood La level unit is millimole per litter. The timeline and data collection process are shown in Fig. 1.

The HR response of the subjects and the intensity of the match were monitored through HR telemetric system (HOSAND TM200, Hosand Technology, Verbania, Italy) before, during and after the match. HR was recorded in beats per minute (bpm) every 5 s throughout the study to get the mean and peak HRs of the subjects. Resting heart rate was measured before the warm-up in a supine position. All players wore a transmitter chest belt (T31 Transmitter; Polar, Alexandria, Australia) during the study protocol^21^. The match period was divided into 12-time frames, just as in the blood La sample collection. Thus, while the data of mean HR involve a time period, the values of mean blood La involve only a single moment in this period.

### Statistical analysis

In the study, the data including the subjects’ HR and blood La responses, time spent in percentages of HR_max_ and time spent in time slots set in a match were given. The data were expressed as mean ± SD. The descriptive statistics and the significance test between the groups were analysed by using the SigmaPlot version 11.0 (from Systat Software, Inc., San Jose, California, USA) software. Shapiro–Wilk normality test was used to determine whether the data showed a normal distribution. The distribution of the data was normal. Holm–Sidak test was used in order to determine the HR and La differences between the player positions. Confidence intervals (95% CI) were calculated and presented where appropriate. One-way analysis of variance was classified using the partial eta squared (η_p_^2^) according to following scale: small = 0.01, medium = 0.06, large = > 0.14^22^. The significance level was determined as p < 0.05.

### Ethics approval and consent to participate

The study was conducted according to the guidelines of the Declaration of Helsinki and approved by the Gazi University Review Board (No: 2022-101). Written informed consent was obtained from the participants to publish this paper.

## Results

**Figure 2:**
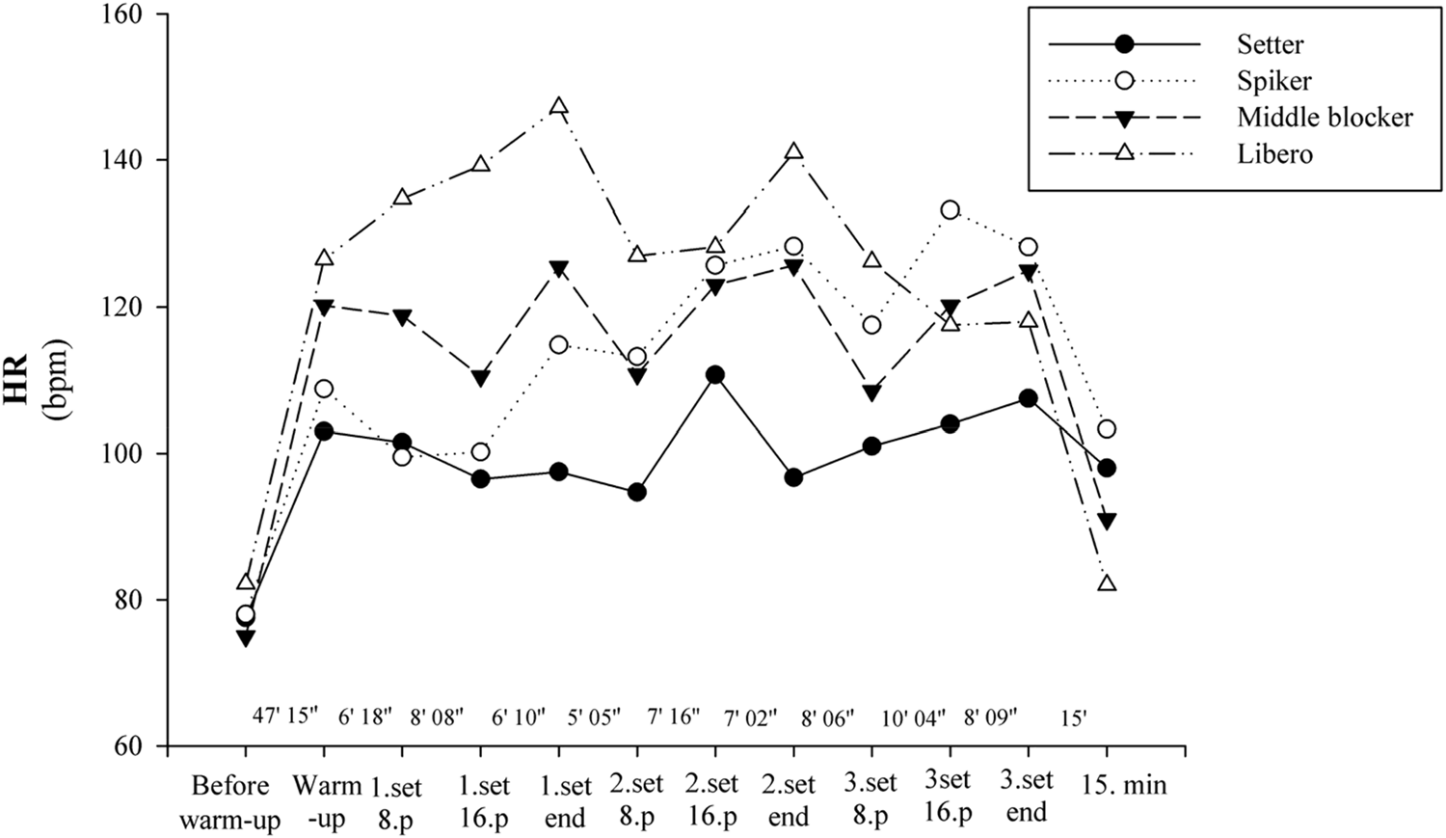
HRs according to playing positions before, during and after volleyball match.

The mean total duration of three matches is 135 min and 16 s. The mean duration of the sets is 20, 19 and 26 min, respectively. The warm-up of the matches lasted approximately 47 min. The athletes completed the volleyball match with mean HR as 117.2 ± 13.9 bpm (95% CI = 112.6–121.7) and mean blood La 2.7 ± 1.2 mmol/L (95% CI = 2.3–3.1). While the mean HR before the warm-up was 75–82 bpm, an increase was observed together with the warm-up (mean 103–126 bpm). The mean HRs throughout the match were measured as setters 101.3 ± 5.2 bpm (95% CI = 97.3–105.3), spikers 116.9 ± 11.9 bpm (95% CI = 107.8–126.1), middle blockers 118.8 ± 6.6 bpm (95% CI = 113.7–123.9), liberos 130.6 ± 9.8 bpm (95% CI = 123.1–138.1). The HRs were significantly different between positions, except spikers vs. middle blockers (setter vs. libero, F = 16.388, η_p_^2^ = 0.969 [large], p = < 0.001; setter vs. middle blocker, F = 16.168, η_p_^2^ = 0.966 [large], p = < 0.001; setter vs. spiker, F = 16. 227, η_p_^2^ = 0.966 [large], p = < 0.001; libero vs. spiker, F = 16.184, η_p_^2^ = 0.974 [large], p = 0.004, libero vs. middle blocker, F = 16.124, η_p_^2^ = 0.974 [large], p = 0.007, Table 2). During the match, setters have the lowest and liberos the highest HR (Fig. 2).

**Table 2:** HR and blood La responses by playing positions before, during and after volleyball match.

	Time	Setter (*n* = 6)				Spiker (*n* = 6)			
		HR (bpm)	Max HR	Min HR	La (mmol/L)	HR (bpm)	Max HR	Min HR	La (mmol/L)
Before warm-up	–	77.5 ± 12.4	–	–	1.9 ± 0.7	78.0 ± 12.9	–	–	1.8 ± 0.8
Warm-up	47′ 15″	103.0 ± 18.1	165	48	1.7 ± 0.8	108.8 ± 22.9	182	49	2.1 ± 1.1
1st set 8th point	6′ 18″	101.5 ± 32.3	144	53	2.3 ± 1.2	99.5 ± 41.3	171	48	4.7 ± 3.1
1st set 16th point	8′ 08″	96.5 ± 18.6	137	58	2.5 ± 0.8	100.2 ± 36.4	153	48	3.4 ± 2.9
1st set end	6′ 10″	97.5 ± 10.9	121	85	1.5 ± 0.3	114.8 ± 32.1	171	67	1.4 ± 0.5
2nd set 8th point	5′ 05″	94.7 ± 28.3	142	48	1.9 ± 0.9	113.2 ± 46.6	211	56	2.1 ± 1.2
2nd set 16th point	7′ 16″	110.7 ± 39.9	155	48	2.9 ± 1.4	125.7 ± 44.8	212	49	2.1 ± 1.2
2nd set end	7′ 02″	96.7 ± 28.8	148	52	3.1 ± 1.5	128.3 ± 45.1	189	48	2.0 ± 1.1
3rd set 8th point	8′ 06″	101.0 ± 31.8	135	56	2.5 ± 1.1	117.5 ± 33.5	183	74	1.6 ± 0.7
3rd set 16th point	10′ 04″	104.0 ± 20.0	134	57	2.4 ± 0.4	133.2 ± 41.7	211	74	2.6 ± 1.5
3rd set end	8′ 09″	107.5 ± 26.5	136	50	2.2 ± 1.0	128.2 ± 23.1	211	67	3.0 ± 2.7
Recovery	15'	98.0 ± 24.2	131	48	2.1 ± 0.9	103.3 ± 12.4	181	55	2.1 ± 1.9
Mean		101.3 ± 5.2	140.7 ± 12.1	54.8 ± 10.7	2.3 ± 0.5	116.9 ± 11.9	188.6 ± 20.2	57.7 ± 10.7	2.5 ± 1.0

	Time	Middle blocker (*n* = 6)				Libero (*n* = 6)			
		HR (bpm)	Max HR	Min HR	La (mmol/L)	HR (bpm)	Max HR	Min HR	La (mmol/L)
Before warm-up	–	75.0 ± 8.3	–	–	2.5 ± 0.9	82.2 ± 3.8	–	–	2.5 ± 1.1
Warm-up	47′ 15″	120.2 ± 26.4	192	53	2.5 ± 0.9	126.5 ± 23.1	211	48	2.1 ± 0.7
1st set 8th point	6′ 18″	118.8 ± 42.5	205	50	4.3 ± 4.0	134.7 ± 41.9	183	65	1.9 ± 0.6
1st set 16th point	8′ 08″	110.5 ± 36.7	183	69	2.7 ± 1.1	139.2 ± 16.1	168	88	2.0 ± 0.6
1st set end	6′ 10″	125.5 ± 32.2	196	82	1.7 ± 0.5	147.2 ± 13.1	172	108	1.9 ± 0.6
2nd set 8th point	5′ 05″	110.8 ± 31.6	169	73	2.7 ± 1.0	127.0 ± 37.3	178	69	2.4 ± 1.1
2nd set 16th point	7′ 16″	123.0 ± 26.4	184	73	4.7 ± 1.1	128.2 ± 25.6	174	74	4.9 ± 4.7
2nd set end	7′ 02″	125.7 ± 31.8	186	78	3.2 ± 2.7	141.0 ± 30.6	179	86	7.0 ± 5.2
3rd set 8th point	8′ 06″	108.5 ± 26.1	142	63	3.6 ± 1.7	126.2 ± 30.5	164	82	1.6 ± 0.6
3rd set 16th point	10′ 04″	120.3 ± 27.6	180	56	1.6 ± 0.4	117.5 ± 30.3	153	66	1.9 ± 0.9
3rd set end	8′ 09″	125.0 ± 18.6	162	50	3.0 ± 2.8	118.0 ± 33.9	166	49	2.0 ± 1.7
Recovery	15'	91.0 ± 15.4	153	57	2.4 ± 0.9	82.0 ± 12.2	114	51	1.5 ± 0.9
Mean		118.8 ± 6.6	177.5 ± 19.1	64 ± 11.6	3.0 ± 1.0	130.6 ± 9.8	169.3 ± 23.4	71.5 ± 18.7	2.8 ± 1.8

Data are shown as mean ± SD. *HR* heart rate, *La* blood lactate.

**Figure 3:**
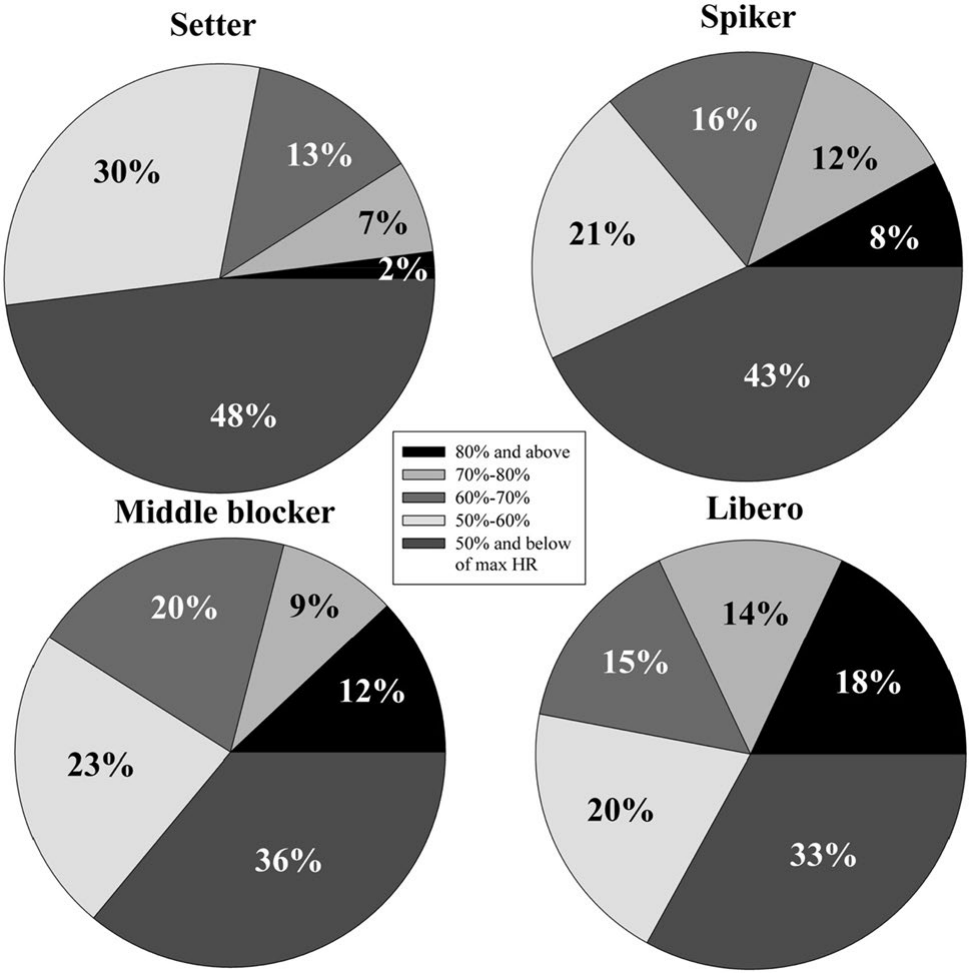
The time spent by the volleyball players in the determined ranges of their HR_max_ before, during and after the volleyball match (%).

Considering the positional assessment; setters have the HR of 80% or above of their HR_max_ in 1.9% of the total match duration and other positions respectively, spikers 8.2%, middle blockers 12.3% and liberos 17.9%. In addition, players have the HR of 60–80% of HR_max_ as setters 43%, spikers 28%, middle blockers and liberos 29% during the match duration. On the other hand, it was determined that setters, spikers, middle blockers and liberos completed the match in the HR range of 50–60% and below 50% HR_max_ in 78%, 64%, 59% and 53% of the total match duration, respectively (Fig. 3).

The blood La levels of the athletes exhibit differences depending on the progress and intensity of the match, technical movement frequencies (such as spiking, serving etc.) and resting periods (time-outs and routine substitutions e.g. middle blockers-liberos). While blood La levels were mostly 1–2–3 mmol/L, 4 mmol/L and above was reached by the spikers in the 1st technical time-out of the 1st set (4.7 mmol/L), by the middle blockers in the 1st technical time-out of the 1st set (4.3 mmol/L) and in the 2nd technical time-out of the 2nd set (4.7 mmol/L), by the liberos in the 2nd technical time-out of the 2nd set (4.9 mmol/L) and at the end of the 2nd set (7.0 mmol/L). The setters never reached 4 mmol/L and above blood La before, during and after the match. The blood La levels between the groups were not found statistically significant (Table 2).

## Discussion

The objective of this study was to determine the HR and blood La responses in elite female volleyball players before, during, and after the match according to playing positions. To the authors’ knowledge, this is the first study in this sample size to report the physiological (i.e. HR blood La) responses of volleyball players during a match and to include all playing positions. The main results of the present study were: (a) HR was statistically different between all playing positions except middle blockers vs. spikers (p < 0.05); (b) the blood La levels between the groups were not statistically different; (c) setters have the HR of 80% or above of their HR_max_ in 1.9% of match play and other positions respectively spikers 8.2%, middle blockers 12.3% and liberos 17.9% during the match; (d) the highest mean blood La level is in the liberos (7.0 ± 5.2 mmol/L, after the 2nd set 2nd half), the lowest mean blood La level is in the setters (range between 1.5–3.1 mmol/L).

As a result of the systematic review, two studies investigating HR and blood La responses in female volleyball players during the match were found^23,24^. Although there are more studies on male volleyball players, the mean HR range was between 105.3 ± 12.8–159 ± 6 bpm and mean blood La level was determined 2.8 ± 0.4 mmol/L in a limited number of female volleyball players (Table 3). Some studies reveal the HR and blood La monitoring in trainings, some in the matches, and some in both. During the training and/or the match, the HR and blood La levels exhibit differences depending on the positions.

**Table 3:** A summary of scientific researches about HR and blood La of volleyball players.

Authors (year)	Subjects	Gender	Training/Match	HR (bpm)	Blood La
Blair (2014)^23^	14 university level volleyball players (*n*_*libero*_: 2, *n*_*middleblocker*_: 3,* n*_*spiker*_: 4,* n*_*setter*_: 2)	F	Pre-season volleyball tournament in 5 matches, in 6–2 system	Mean HR for libero: 133 ± 14, middle blocker: 133 ± 20, spiker: 159 ± 6, setter: 157 ± 7 Spikers have the HR_max_ (mean 182 ± 5)	Not included
Duarte et al. (2019)^25^	15 professional volleyball players	M	Technical and tactical training	90–100% of HR_max_ has been reached in 17% of defence training (max percent of all training types at this zone, followed by block training with 15%) 70–80% of HR_max_ has been reached in 35% of tactical training	Not included
Gabbet et al. (2006)^2^	Twenty-six junior volleyball players (mean age, 15.5 ± 0.2 years)	NM	Skill-based training	HR 40–70% of HR_max_ and 138 ± 2 of mean HR has been reached in 57.4 ± 3.6% of the training time	Not included
González et al. (2005)^9^	30 players from 10 teams, only middle blocker and libero players were included	M	Match	Mean and HR_max_ values between the central players and the libero were found statistically different (p < 0.01)	Middle blocker: 4.12 mmol/L vs Libero: 3.23 mmol/L 59.1% samples refer to values of less than 4 mmol/L
Karaca et al. (2018)^24^	Healthy female competitive volleyball players (n = 12), and a control group (n = 12), aged 16–22 years old	F	Training matches	HR were significantly higher in the control group (132.9 ± 16.5 vs. 105.3 ± 12.8)	Study group: 2.8 ± 0.4 mmol/L Control group: 3.7 ± 1.0 mmol/L
Kasabalis et al. (2005)^10^	Totally thirty-six elite volleyball players (juniors (15-16yrs, n = 20) and seniors (18-25yrs, n = 16)	M	In laboratory test (max VO_2_) and training and match	In competition: HR range in junior and senior respectively 152–191 and 175–193 (p < 0.01) HR_max_: 182.3 ± 5.2 vs. 170.9 ± 12.7 (p < 0.01)	Blood La concentration range in junior and senior respectively 2.36–10.66 and 2.31–9.77 mmol/L
Kawczyński et al. (2010)^26^	National Junior Team volleyball players (n = 14)	M	Match	Not included	With the mean range of 1.1–1.7 mmol/L
Mroczek (2007)^15^	10 volleyball players	NM	Match	Mean HR during the game ranged from 90 to 149 bpm, with min. 65 bpm during rest and max 199 bpm	Not presented
Mroczek et al. (2013)^27^	National Junior Team volleyball players (n = 14)	M	Match	Mean HR in Set 1: 138.1 ± 14.7 Set 2: 135.3 ± 15.9 Set 3: 136.8 ± 18.1 Set 4: 132.6 ± 16.8	Mean blood La in Set 1: 1.7 ± 0.4 Set2: 1.5 ± 0.5 Set 3: 1.4 ± 0.2 Set 4: 1.3 ± 0.2
Rajkumar (2020)^28^	12 inter-collegiate volleyball players were	M	Match_in dehydration conditions	Pre-match HR mean: 73.7 ± 4.7 Post-match HR mean: 90.5 ± 16.1	Not included

In gender column, *F* female, *M* male, *NM* not mentioned. Data shown as mean ± SD. *HR* heart rate, *La* lactate.

Within the samples taken from the female volleyball players, Karaca et al.^24^ have determined that the volleyball matches create moderate physiological stress (105.3 ± 12.8 bpm; 2.8 ± 0.4 mmol/L). The physiological stress level determined during the match is an essential indicator that trainers and conditioners can use in training. For example Gabbett et al.^2^ have determined that in volleyball trainings, in the majority of the training time (57.4 ± 3.6%) the HR progresses at very low intensity (40–70% of HR_max_) and the mean HR is 138 ± 2 bpm. While HR response changes were observed in technical (80% of HR_max_) and tactical training (70% and 90% of HR_max_^25^) the findings indicate that; besides the volleyball is a sport that requires especially dynamic and explosive power, it uses more phosphocreatine from the anaerobic glycolytic system, and towards the end of the match, the aerobic capacity becomes a more prominent factor than the anaerobic power sources with high-level energy^10^.

On the other hand, studies that examine between positions provide more specific information. In one of these studies, the highest mean HR was in the spikers, while the setters spent most of the match with 80% predicted HR_max_. And the liberos had the lowest mean HR (mean HR respectively, libero (n = 2): 133 ± 14, middle blocker (n = 3): 133 ± 20, spiker (n = 4): 159 ± 6, setter (n = 2): 157 ± 7, in 6–2 playing system). Following the long resting periods, the middle blockers had a high HR “on the court”^23^. In this study, which is similar to our research design—such as gender, positional differences, match responses—mean HR was found to be higher than in our research. The reason for this is that the researcher preferred the 6–2 game system—5–1, which is the most widely used game system, was applied in our research—and the highest HR was observed in the setters, as a result of the setter taking the role of opposite hitter in the front row and setter in the back row position in this system. This means an extra load for the setter. Therefore, these findings are not similar to our study’s findings. Another study^9^, reports that middle blockers and liberos differentiate between each other in terms of HR and La responses. As expected, it was determined that both player positions have a decrease in HR while they were off the court. In addition, the blood La of the middle blockers is significantly higher than the liberos (4.12 mmol/L vs 3.23 mmol/L). Liberos have a relatively shorter time on the bench, so their HR decrease was not as drastic as determined by middle blockers. Another finding in the study is the high lactate concentration which is being reached by the players. 40.9% of all the blood La concentrations corresponded to values higher than 4 mmol/L, and 2.8% were above 8 mmol/L. Therefore, researchers associated volleyball with high blood La production despite the intermittent game structure. Since it was a study conducted on male subjects, it is expected that the values they found were higher than those obtained in our study.

Mroczek^15^ found that the mean HR of volleyball players was between 90 and 149 bpm, although the highest mean HR in a volleyball match was 149 bpm (with the range between 89 and 199 bpm) in his study completed with a small number of subjects. The fact that the setters have the lowest HR among the positions is consistent with the findings of our study. In a study in which only blood La levels were included, the blood La levels of young volleyball players increased significantly at the end of each set compared to the pre-game (pre-game 1.1 mmol/L, 1st set 1.7 mmol/L, 2nd set 1.5 mmol/L, 3rd set 1.4 mmol/L, 4th set 1.3 mmol/L^26^) According to Künstlinger et al.^29^, volleyball is characterized with a low concentration of La (2.54 ± 1.21 mmol/L) during and after the match. This indicates that the increase in free fatty acids indicates that energy is provided mainly by the breakdown of creatine phosphate during short periods of activity (9 s), while aerobic pathways restore energy sources during rest periods (12 s). In summary, we can say that the findings in the studies which make comparisons between positions are changeable, and no common result has been encountered. In summary, the findings in the studies conducted playing position comparisons were different and no common result was found.

## Conclusion

We have reported the physiological responses of volleyball players during the match. The strongest and at the same time the main limitation of this study is the sample size. Although a study with a sample size like this current study has not been studied before, reaching a larger sample size is recommended for future research. Our results showed HR and blood La levels differ between playing positions before, during and after a volleyball match. And the players who complete the match with highest intensity are the liberos. In addition, our research supports that volleyball players need high levels of oxidative capacity and creatine phosphate and glycolytic energy systems^4^, during a match, but low (very light to light < 57% to < 64%) intensity activity^30^ was observed during more than half of the match. In volleyball trainings, the loading intensity can be kept at 60% of the HR_max_ or below during 50–60% of the training time, and it can be provided as 80% of the HR_max_ or above during 10% (as mean value) of the training time. In addition to the necessity of including athletes in training at specific loading intensity according to their positions, it is very important to apply high-intensity activities with long rest intervals. While performing these applications, factors such as the training periodization (such as pre-season, in-season, inter-season), the purpose of the training, the readiness of the athletes, and the upcoming competition day should not be neglected. In order to achieve a more enlightening result, it is recommended to carry out simultaneous time-motion analysis and movement profile analysis in addition to these measurements performed in the competition environment in future studies.

## Data Availability

The data presented in this study are available on website: https://osf.io/y2ugd/ with Identifier: 10.17605/OSF.IO/Y2UGD.
